# Measurement of Elevated IL-37 Levels in Acute Ischemic Brain Injury: A Cross-sectional Pilot Study

**DOI:** 10.7759/cureus.1767

**Published:** 2017-10-11

**Authors:** Atif Zafar, Asad Ikram, Dinesh V Jillella, Duraisamy Kempuraj, Mohammad Moshahid Khan, Saif Bushnaq, Harold Adam, Santiago Ortega-Gutierrez, Syed A Quadri, Mudassir Farooqui, Asgar Zaheer, Enrique C Leira

**Affiliations:** 1 Cerebrovascular Medicine, University of New Mexico School of Medicine, Albuquerque, New Mexico, USA; 2 Neurology, School of Medicine, University of Missouri, Columbia, Mo, Usa; 3 Neurology, University of Tennessee Health Science Center Memphis; 4 Neurology, University of Iowa Hospitals and Clinics, Iowa City, Ia, Usa; 5 Neurosurgery, California Institute of Neurosciences; 6 Neurosciences, Oklahoma; 7 Neurology, University of Missouri

**Keywords:** interleukin-37, stroke, acute ischemic brain injury, post-stroke inflammation, cytokine, pro-inflammatory responses

## Abstract

Interleukin (IL)-37 is a new member of the IL-1 cytokine family with a defined role as a negative feedback inhibitor of proinflammatory responses. IL-37 has yet to be evaluated in non-immune-mediated neurological diseases, such as ischemic or hemorrhagic strokes. This study aimed to measure urine and serum IL-37 levels in patients with ischemic stroke. Twelve patients consented for our study. Two sets of serum and urine samples were obtained and analyzed, one upon admission to the hospital and the second the next morning. The trends in serum levels of IL- 37 in six stroke patients and the trends in the urine levels of eight stroke patients were measured by real-time polymerase chain reaction (RT-PCR) and enzyme-linked immunosorbent assay (ELISA). Our pilot study showed IL-37 levels in urine in stroke patients ranging between 210 and 4,534. Serum IL-37 levels were in the range of 44 - 5,235 in patients with ischemic stroke. Three patients who presented within three hours of stroke onset had IL-37 serum levels of 2,655 pg/ml, 3,517 pg/ml, and 5,235 pg/ml, respectively. In all others, it ranged much less than that, with the trend of delayed presentation giving lower IL-37 levels. The study shows a rather stable early elevation of serum IL-37 levels post-ischemic stroke. IL-37 plays a certain role in mediating post-stroke inflammation with a significant increase in serum levels of this novel cytokine observed in ischemic stroke patients. Further large-scale studies need to be done to establish its definite role. A prospective "CRISP" trial is registered with the ClinicalTrials.gov (Identifier: NCT03297827) to determine the role of IL-37 in modulating post-stroke inflammation.

## Introduction

The basic mechanism involved in ischemic stroke indicates that endothelial dysfunction, along with oxidative stress and inflammation, characterize a key step in the cerebral ischemia/reperfusion (I/R) injury [1-2]. The inflammatory response involving cytokines, adhesion molecules, chemokines, and leukocytes that occurs at the blood-endothelium interface of the cerebral vessels [3-4] plays a critical role in the pathogenesis of tissue damage in cerebral infarction [5-6]. Several cytokines, such as interleukin (IL)-6, IL-1, IL-8, and tumor necrosis factor alpha (TNF-α), have been linked with central nervous system (CNS) injury and ischemic injury [3-5]. IL-37 is a relatively new member of the IL-1 cytokine family, which functions as a fundamental inhibitor of innate inflammation, but its exact role in the brain remains vague [7]. It has been investigated in patients with systemic autoimmune and rheumatologic diseases, with active disease correlating with higher IL-37 levels [8-18]. Its role in neuroimmunological diseases, such as multiple sclerosis (MS) and Guillain-Barre syndrome (GBS), has also been studied [19-20]. IL-37 has yet to be evaluated in other non-immune neurological pathologies like ischemic or hemorrhagic strokes. Although some animal model studies have been carried out to determine the effects of this relatively new cytokine on brain inflammation induced by brain ischemia/reperfusion (I/R), there have been no human studies done to establish its elevation and significance in stroke patients. This study aimed to measure the urine and serum IL-37 levels in patients with acute ischemic stroke. IL-37 is a newly identified member of the cytokine family, and this is the first ever-pilot study performed to understand its role in acute ischemic stroke patients.

## Materials and methods

A total of 12 patients who were admitted with ischemic stroke to the Department of Neurology were enrolled in the study. The institutional review committee approved the study at the University of Iowa Hospitals and Clinics, and a written informed consent was obtained from individual participants before enrollment. Experienced neurologists evaluated all the patients, and the diagnosis of acute stroke was made based on MRI (magnetic resonance imaging) of the brain. Patients who had a history of autoimmune diseases, such as multiple sclerosis (MS), rheumatoid arthritis (RA), inflammatory bowel disease (IBD), and diabetes mellitus type-I (DM-I), or chronic inflammatory diseases, such as metabolic syndrome, diabetes mellitus type-II (DM-II), chronic cardiovascular disease, malignancy, history of infection within the last three months, or history of heavy smoking, were excluded from the study.

Specimen collection and preparation 

Two sets of serum and urine samples were obtained and analyzed from individual participants; one was obtained upon admission to the hospital, and the second sample was collected the next morning after overnight fasting. Both sets of blood samples (3-5 ml) were collected from peripheral veins according to the routine puncture method and were done for clinical purposes. The stored blood and urine samples that were drawn but not used by lab-pathology for clinical care were utilized for the study. They were subjected to centrifugation, and the resulting plasma and cerebrospinal fluid supernatants were stored at −80∘C. All procedures were followed in accordance with the institutional guidelines.

Measurement of IL-37

The serum and urine levels of IL-37 in our patient population were measured by real-time polymerase chain reaction (RT-PCR) and enzyme-linked immunosorbent assay (ELISA). These were determined using a commercially available human IL-37 ELISA kit (R&D System Human Premixed Multi-Analyte Kit) (AdipoGen Life Sciences, Liestal, Switzerland), according to the manufacturer's instruction. Individual plasma and urine samples were diluted at 1:1 and tested in triplicate by ELISA. The concentrations of plasma IL-37 of individual samples were determined using the standard curve established and using the recombinant IL-37 provided.

## Results

**Figure 1:**
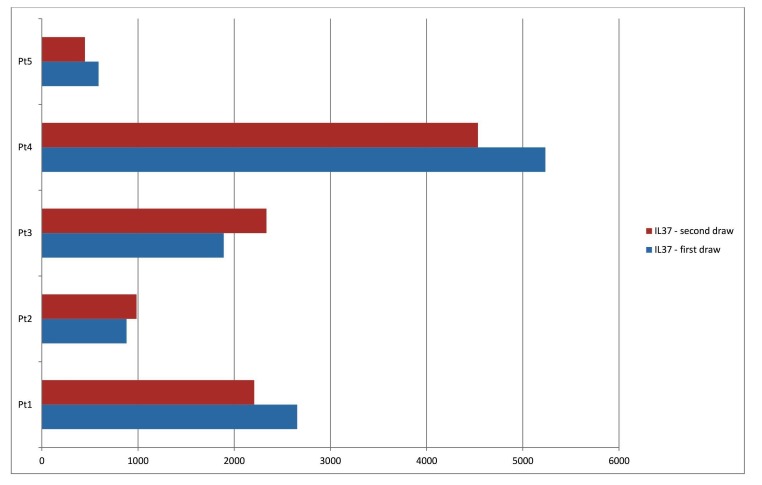
Serum IL-37 level in acute stroke patients at the time of admission (blue bars) and after 24 hours. IL-37: Interleukin-37

Twelve patients (eight males) with the median age of 62.5 years were enrolled in the study. The serum levels of IL-37 in six stroke patients and urine levels in eight stroke patients were available, measured by RT-PCR and ELISA. Our pilot study showed IL-37 levels in urine in ischemic stroke patients ranging from 210 - 4,534 and serum IL-37 levels in the range of 44 - 5,235. Ten patients had at least one value of serum IL-37 available ranging from 44 - 5,235 pg/ml as shown in Table 1. Three of them presented acutely within three hours of stroke onset with IL-37 serum levels being 2,655 pg/ml, 3,517 pg/ml, and 5,235 pg/ml (Figure 1). There was one patient with dissection as the cause of a small stroke where the IL-37 level was negligible. A patient who presented at least one week out from stroke onset and another presenting as a transfer from an outside hospital about one month out from his stroke had levels of 44 and 333, respectively. The other cases with lab collection from stroke onset between 3 - 24 hours had IL-37 levels in the range of 500 - 1,900 pg/ml.

**Table 1:** Demographics, Stroke Characteristics, and Serum IL-37 Levels of the Patients IL-37: Interleukin-37; NIHSS - National Institutes of Health Stroke Scale; F: female; M: male; N/A: not available; CC: corpus callosum; CR: corona radiata

Pt #	IL-37 (pg/ml)	AGE	GENDER	TIME FROM ONSET TO PRESENTATION (in minutes)	NIHSS ON ADM	STROKE LOCATION	DIMENSIONS OF STROKE (mm)
4	5,235	48	M	145	0	Peri-rolandic	16.9 x 11.3
9	3,517	70	M	60	9	Parietal/temporal	N/A
1	2,655	78	M	165	3	Temporal/occipital	Temporal = 7.5 x 9.4; occipital = 7.5 x 5.6
7	2,515	47	M	360	0	Medial thalamus	6 x 6 mm
3	1,891	82	M	513	2	Frontal	N/A
2	881	79	F	385	17	Frontal/anterior cingulate gyrus	N/A
10	614	60	M	315	4	Corona radiata	7.5 x 3.8
8	590	46	F	2,400	6	Cerebellar	54.5 x 43.2
6	333	58	F	N/A	2	Multiple	15 x 9.4 genu of CC, 15 x 13 CR, small watershed
5	44	64	M	5,880	1	Cerebellar	9 x 7.2

**Figure 2:**
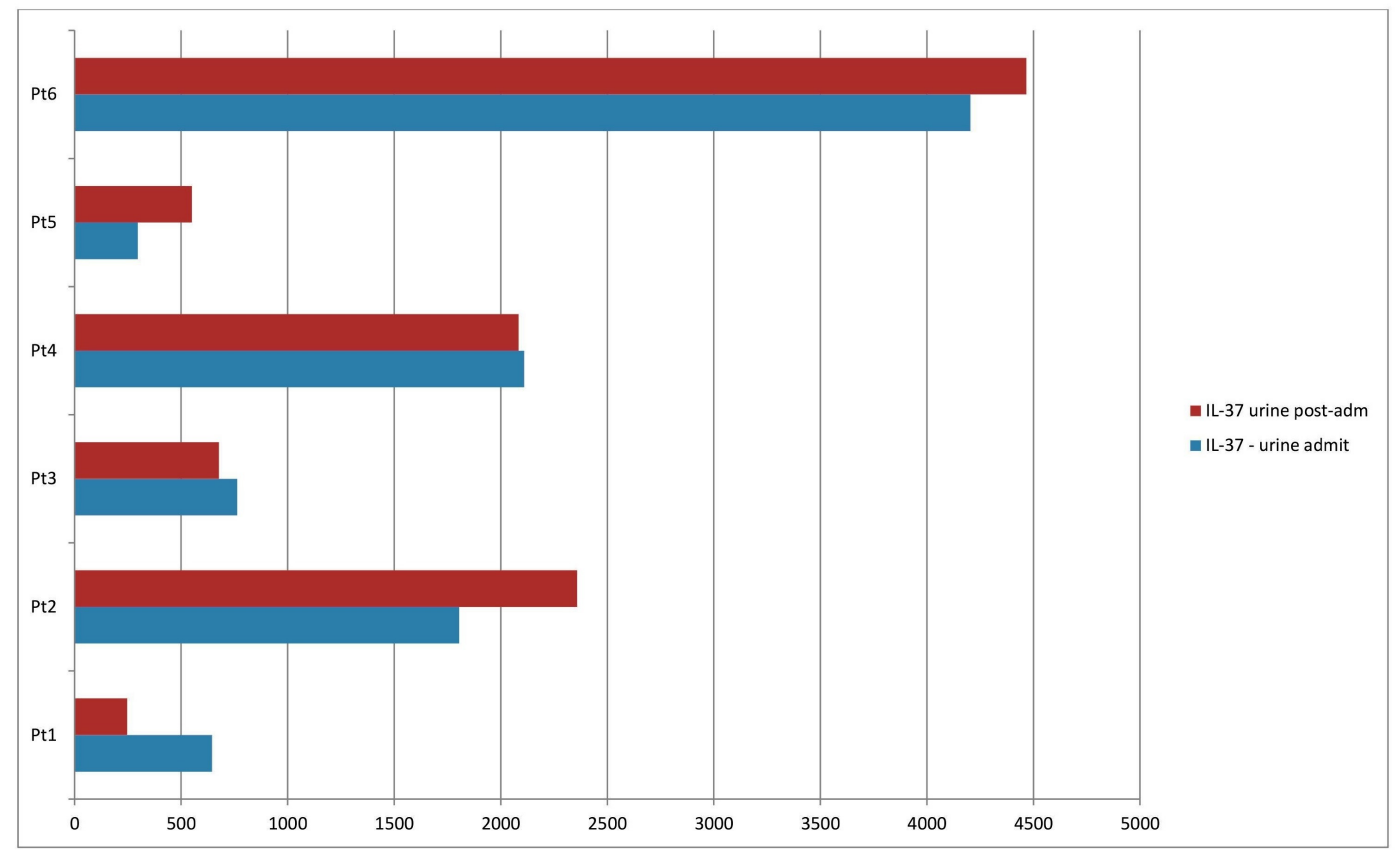
Urine IL-37 level in acute stroke patients at the time of admission (blue bars) and after 24 hours. IL-37: Interleukin-37

Only nine patients had at least one urine specimen available. Similar to the serum findings, the three patients presenting typically within three hours of onset, categorized as acute, had urine IL-37 levels in ranges higher than 3,000 (Figure 2). In six other urine samples, it ranged much less than that, with the trend of delayed presentation giving lower IL-37 urine levels. Out of eight patients, half showed an uptrend, and the rest showed a downtrend.

Out of six patients who presented within four and a half hours, five received intravenous tissue plasminogen activator (iv-tPA) with eventually some infarct noted on imaging in all the six patients. There were no clear differences found in the IL-37 levels in patients with respect to the IV-tPA administration status. Three of the 12 patients who underwent an interventional procedure, with one of the dissection cases needing three stents in the ICA, had no urine or serum IL-37 presence. The other two patients who underwent thrombectomy did have IL-37 levels in the expected range.

## Discussion

The increased expression of several pro-inflammatory cytokines, such as TNF-α and IL-8, post-stroke has previously been established [3-5]. The role of these cytokines as potential therapeutic targets for prevention of ischemia-reperfusion injury and in the treatment of acute ischemic stroke has been brought into the light [5]. IL-37, formerly termed IL-1 cytokine family member-7, is produced by various types of cells, including natural killer (NK) cells, monocytes, activated B cells, and keratinocytes [8, 10]. IL-37 broadly reduces innate inflammation as well as acquired immunity [10]. It is a negative feedback inhibitor of pro-inflammatory responses as it has been shown to reduce expression of several pro-inflammatory cytokines in cell cultures and various inflammatory disorders [8-18]. It was discovered by in silico research of human databases [8, 10, 18]. The cytokine is similar to IL-1 and IL-33, in that it is found in the cell nucleus where it functions in transcription [21]. Translocation of IL-37 to the nucleus likely involves SMAD3, which is a component of the transforming growth factor beta (TGF-β) anti-inflammatory signaling pathway [22]. Upon cell death and loss of membrane integrity, the IL-37 precursor exits from the cell where it binds to the IL-18 receptor alpha chain, resulting in reduced inflammation. It has been investigated in patients with systemic lupus erythematosus (SLE), Graves’s disease, ankylosing spondylitis (AS), rheumatoid arthritis (RA), hepatic ischemia, and cardiac ischemic conditions, including acute coronary syndrome (ACS) and MI, with active disease correlating with higher IL-37 levels [8-18, 23-25]. In two different studies performed in GBS and MS patients, IL-37 levels were elevated in patients having these disease entities compared to control groups (range: 65 - 130 pg/ml) [19-20]. Our study shows a rather stable elevation of IL-37 levels post-ischemic stroke, which (if compared to available data from other studies) is three to ten times elevated after acute ischemic stroke with an uptrend noted in the first few days [19-20]. This suggests that IL-37 plays some role in mediating post-stroke inflammation based on the significant rise in serum and urine levels. An additional interesting inference noted in GBS patients was that there was a decline in IL-37 levels by four weeks in those who responded to treatment and showed clinical neurological improvement compared to patients who showed a neurological decline and had persistently elevated IL-37 levels [20]. In our study, five patients received iv-tPA with eventually some infarct noted on imaging. We did not find any clear differences in patients who received tPA versus who did not. Three of the 12 patients underwent an interventional procedure with either a thrombectomy or a stent placement. One of them, with a carotid dissection requiring three stents in the internal carotid artery, demonstrated no urine or serum IL-37 presence. The other two patients who underwent thrombectomy did show increased IL-37 levels in the expected range. The role of IL-37 in cardiovascular diseases has recently surfaced. In one study performed on mice models, IL-37 seemed to improve cardiac function if given after acute myocardial infarction (MI) [25]. In a human study on patients admitted to hospitals after acute ST- elevation MI (STEMI), plasma IL-37 levels were found to be elevated in both hypertensive and non-hypertensive patients [26]. There were no significantly different findings in two of the 12 patients with diabetes in our study. Similarly, patients with varying LDL levels and blood pressure did not seem to have any effect on the IL-37 levels in our small study. One major limitation of this study is the absence of a control group. Prior studies with healthy volunteers as a control group have consistently shown IL-37 plasma levels around or less than 65 pg/ml with maximum normal levels on ELISA approximated at 130 pg/ml. The role of IL-37 in stroke has remained elusive so far and lacks human studies. From a prior animal study, IL-37 has been shown to be involved in the mechanism of cerebral ischemia-reperfusion injury by participating in the process of injury through the decrease of proinflammatory cytokines: TNF α, IL-6, IL-1b, monocyte chemoattractant protein-1 (MCP-1), and macrophage inflammatory protein-1 (MIP-1) [27]. Ours is the first ever reported study measuring and trending IL-37 levels in human plasma after an acute ischemic stroke. Our pilot project confirms the rise of IL-37 levels post-stroke in humans. Given these affirming results, the next step would be to look at a larger patient pool of acute stroke patients and comparing them to healthy controls and investigate the correlation of infarct size, neurological deficits, and improvement in their motor function with the IL-37 levels. The multi-fold increase in the level of serum and urine IL-37, as is evident from our initial results, indicates a possible key role of this novel cytokine in post-stroke pathology as a modulator of brain inflammation and warrants further investigation in human populations. Besides its vital role in innate immunity, it may have important translational implications as a potential new target for both clinical preventive and therapeutic strategies in patients suffering from a cerebral ischemia/reperfusion injury.

## Conclusions

In summary, elevated levels of IL-37 in the acute systemic inflammatory disorders, as well as neurological diseases, makes it an intriguing novel cytokine with potential diagnostic, prognostic, and therapeutic roles. Whether this surge in IL-37 is a factor limiting further stroke-related inflammation or something that causes tissue damage is difficult to infer from this small pilot project. Large-scale studies are needed in the future to further elaborate and understand its role in stroke. Based on these findings, our group has recently started a prospective clinical trial with the ClinicalTrials.gov (Identifier: NCT03297827) and acronym "CRISP" trial, which we hope will shed some light on the role of IL-37 in modulating post-stroke inflammation.
